# Understanding the School Food Environment and Anthropometric Indicators of Schoolchildren: A Census-Based, Cross-Sectional Study Using Primary Data in Rural Brazil

**DOI:** 10.3390/ijerph23040427

**Published:** 2026-03-29

**Authors:** Raisa Pessini Pizetta, Maria Clara Barcelos de Aquino, Suzana Souza Caldana, Gabryela Pirovani da Fonseca, Adriana Hocayen de Paula, Wagner Miranda Barbosa, Alberto Caixeta Botelho, Débora Nogueira Lopes, Flávia Vitorino Freitas, Míriam Carmo Rodrigues Barbosa

**Affiliations:** 1 Programa de Pós-Graduação em Nutrição e Saúde (PPGNS), Universidade Federal do Espirito Santo (UFES), Vitória 29047-185, Brazil; mariaclarabarcelos0912@gmail.com; 2 Curso de Nutrição, Departamento de Farmácia e Nutrição (CCENS), Universidade Federal do Espirito Santo (UFES), Alegre 29500-000, Brazil; suzanacaldana0@gmail.com (S.S.C.); gabryelapfonseca@gmail.com (G.P.d.F.); adriana.paula@ufes.br (A.H.d.P.); wagner.barbosa@ufes.br (W.M.B.); flavia.freitas@ufes.br (F.V.F.); 3 Programa de Pós-Graduação em Geociências (PPGGEA), Faculdade de Ciências e Tecnologia da Universidade Federal de Goiás (UFG), Aparecida de Goiânia 74971-451, Brazil; albertocaixeta@gmail.com (A.C.B.); deboranogueira@ufg.br (D.N.L.)

**Keywords:** school food environment, community environment, nutritional status, food retail, dietary pattern, PNAE

## Abstract

**Highlights:**

**Public health relevance—How does this work relate to a public health issue?**

- The study is relevant as it focuses on the food environment, a topic that has been widely investigated in recent years and is recognized as an important social determinant of health.
- The findings contribute to the analysis of how environmental factors may influence child development.

**Public health significance—Why is this work of significance to public health?**

- The study is of great importance to public health as it fills a knowledge gap regarding the school food environment in small-sized municipalities, settings that remain underexplored in scientific research.
- The study provides support for actions aimed at promoting adequate and healthy eating, contributing to the strengthening of preventive strategies from early childhood.

**Public health implications—What are the key implications or messages for practitioners, policy makers and/or researchers in public health?**

- The study provides evidence to support the formulation of public policies in school and community settings, aimed at promoting adequate and healthy eating.
- The findings strengthen the conceptual and empirical basis for future studies, particularly those focused on analyzing the association between the food environment and nutritional status.

**Abstract:**

There is a gap in knowledge regarding the school food environment in small-sized municipalities. Thus, this study aims to analyze the anthropometric status of schoolchildren and the school and community food environments in a small Brazilian municipality. This is a cross-sectional, exploratory, and ecological study conducted in elementary schools and food retail outlets in Jerônimo Monteiro, Espírito Santo, Brazil. Anthropometric indicators were assessed using the students’ weight and height. The school food environment was analyzed by evaluating the National School Feeding Program (PNAE) menu using the IQ-COSAN index, classifying foods brought in lunchboxes and sold at schools according to the Brazilian Dietary Guidelines, and auditing food retailers outside schools using the ESAO-S and ESAO-R instruments. Food establishments were categorized according to the Locais-Nova classification and scored using the Healthy Food Store Index (HFSI) and Healthy Meal Restaurant Index (HMRI). The study included 2 schools and 266 schoolchildren (5–11 years), of whom 33.1% had excess weight. The PNAE menu was classified as “needing improvement,” and 81% of schoolchildren’s lunchboxes contained processed/ultra-processed foods. In the external food environment around schools, low levels of access to healthy foods and predominance of ultra-processed food sales were observed.

## 1. Introduction

The human right to adequate and healthy food is enshrined in Brazil by Law No. 11,346 of 15 September 2006 [1]. This right is extremely important, especially in childhood, given that ensuring adequate and healthy nutrition during this period of life is fundamental for children’s physical and cognitive development [2,3]. However, the current reality of children’s diets does not follow the precepts of Law No. 11,346, as studies have found high consumption of ultra-processed foods (UPFs) by this age group [4,5,6,7].

In Latin America, the consumption of UPFs has had a significant impact on the caloric intake of schoolchildren, representing approximately 18% to 44% of the total energy value of their diet [8]. This scenario is also observed in different regions of Brazil, revealing that UPFs are increasingly present in children’s eating habits [9,10,11]. This reality requires attention, since the consumption of UPFs contributes to the development of type 2 diabetes, hypertension, dyslipidemia, overweight, and obesity [7,12,13].

Given this scenario, it is noteworthy that childhood obesity has emerged as a major problem and challenge for public health in Brazil and worldwide, whose etiology is multifactorial and involves several factors, with lack of access to healthy foods and the prevalence of obesogenic food environments being one of the main points that exacerbate this problem [14,15]. The so-called obesogenic food environment refers to a set of conditions, influences, and food availability that contribute to the occurrence and increase in obesity in the population, as these factors favor the choice of unhealthy foods and excessive calorie consumption [16]. This is harmful to children who are in the phase of developing and consolidating eating habits.

Notably, the formation of children’s eating habits is influenced by different aspects and determined by the eating habits of their family and community, with the school community being a key environment in this formation due to the influence it exerts on food choices and the long period of time children spend there [17,18]. The UN Food and Agriculture Organization (FAO) defines the school food environment as one that encompasses all spaces and conditions in and around schools where food is available for obtaining, purchasing, and consumption, in addition to reiterating the importance of a healthy school food environment that encourages better eating [19]. However, scientific evidence has shown that there is a predominance of establishments focused on the sale of UPFs in the school food environment and in the community environment in which these children live, exposing this population to obesogenic environments that negatively influence food choices [20,21,22,23,24].

Scientific evidence evaluating food supply and the distribution of points of sale in the school food environment, as well as the nutritional status of students, has been growing in recent years and is essential for formulating public policies towards promoting adequate and healthy eating. However, there is still a gap in knowledge regarding the description of these food environments in small and rural municipalities in Brazil, since most of the evidence in the literature is focused on high-income countries and large Brazilian cities [20,25,26,27,28,29,30]. The assessment of the food environment in these municipalities is extremely important, as a considerable part of Brazil’s territory is made up of small-sized municipalities [31].

Thus, this study aims to analyze the anthropometric indicators of schoolchildren and the school and community food environment in a small rural Brazilian municipality in order to contribute to filling the gap in the literature and serve as a basis for policy-making in the field of food and nutritional health and safety, guaranteeing the human right to adequate food.

## 2. Materials and Methods

### 2.1. Study Design

This is a cross-sectional, exploratory, and ecological study that was conducted in food retail establishments and in two municipal primary schools located in the urban center of the municipality of Jerônimo Monteiro (JM) in the state of Espírito Santo (ES), Brazil. The activities related to the study were carried out according to the schedule below (Table 1), with data collection conducted in 2024.

This study is part of a larger project entitled “Ambiente alimentar e a qualidade de alimentos ofertados em torno de escolas do ES” [Food environment and the quality of food offered around schools in ES]. It was approved by the Human Research Ethics Committee of the Universidade Federal do Espírito Santo (UFES) under opinion number CAAE 75806223.6.0000.5060.

### 2.2. Study Region

The municipality of JM is part of the south-central region of the state of Espírito Santo and borders the municipalities of Alegre, Cachoeiro de Itapemirim, Muqui, and Mimoso do Sul. The city is considered small, with a land area of 177.342 km^2^ and a population estimated by the Brazilian Institute of Geography and Statistics (IBGE) at 11,575 inhabitants and a population density of 65.27 inhabitants per square kilometer, with 78% of the population living in urban areas and 22% in rural areas. Its economy is focused on the service sector, commerce, and agricultural activities [32].

### 2.3. Data Collection in the School Environment

Initially, after obtaining approval from the JM Municipal Education Secretariat, initial contact was made with the principals of each school to present the project. Subsequently, visits were made to the schools to learn about and observe the school routine in order to plan for better performance in data collection. Data collection was conducted by a team of researchers composed of students from the Nutrition course, previously trained and supervised by nutritionists, in order to ensure the standardization and quality of the data collection procedures. The data collection activities were carried out during the 2024 school year.

#### 2.3.1. Population and Sample

The population was selected after the signing of the consent form by the Municipal Education Secretariat and the municipal primary schools in the municipality of JM, ES.

The study included students enrolled in grades 1 to 4 of primary education in the morning and afternoon shifts of the two municipal schools located in the urban center of the municipality, whose parents/guardians authorized their participation through the free and informed consent form. The children also gave their consent through the free and informed assent form.

The sample was based on the calculation for a simple random sample. The calculation took into account the total number of schoolchildren (*n* = 657) enrolled from the years 1 to 4 of primary education in municipal schools in JM, ES. An unknown prevalence of 50%, an absolute precision of 5%, and a design effect of 1 were assumed. Finally, an additional 10% was added to account for possible losses. The final sample comprised 265 students, calculated using the virtual calculator “OpenEpi” [33].

#### 2.3.2. Anthropometric Assessment of Students

The students’ body weight and height data were collected for subsequent calculation of the Body Mass Index (BMI). A digital scale with an accuracy of 100 g and a maximum capacity of 150 kg (Oxer Body^®^ 923) was used to measure weight. Height (in centimeters) was measured on a level, rigid surface using a tape measure with a scale of 1 cm and a range of 1 cm to 150 cm. Anthropometric measurements were performed by a pair of researchers previously trained and qualified in standardized measurement procedures. For data collection, the students were taken to a reserved room and released gradually, class by class, accompanied by the teachers responsible, in order to provide greater comfort and safety for the children during the procedure.

Anthropometric measurements were obtained in triplicate, and subsequently the average of the three measurements was calculated for analysis purposes. However, in cases where the first two measurements presented identical values, the third measurement was not performed, considering the consistency of the results obtained in the first two measurements.

BMI was calculated as the ratio between body weight and the square of height (BMI = kg/m^2^). Anthropometric measurements were analyzed using the Antro and AnthroPlus programs, which are specifically recommended by the WHO for the anthropometric assessment of children and adolescents. Based on weight and height, BMI was calculated, and the anthropometric index BMI-for-age (BMI/A) was used for children aged 5 years and older, with classification performed according to reference z-scores derived from the WHO growth curves [34].

#### 2.3.3. Assessment of the Availability of Food and Beverages Offered to Schoolchildren

A qualitative analysis was performed to assess the different food items that schoolchildren had access to during the period they attended school; namely, the theoretical menu prepared by the nutritionist responsible for the National School Feeding Program (PNAE), snacks brought from home by the children, and food products available for purchase within the school environment.

The evaluation of the PNAE’s theoretical menu was carried out over a period of 13 months in 2023 (February to November) and 2024 (February to April), based on a request for a copy of the menus from the municipal executive education secretariat. The tool used for this analysis was the *Índice de Qualidade da Coordenação de Segurança Alimentar e Nutricional* (IQ COSAN—Quality Index of the Food and Nutrition Security Coordination).

The IQ COSAN is an Excel-based tool used to assess the quality of school meal menus under the PNAE, considering aspects such as the frequency and diversity of foods offered, the inclusion of regional foods, and the absence of restricted or prohibited foods. Based on these criteria, points are awarded per day and then a monthly score is generated by calculating the average for each week analyzed. The score can range from 0 and 95 points and classifies the menus as inadequate (0 to 45.9 points), needing improvement (46 to 75.9 points), and adequate (76 to 95 points), so this average score for each month was considered for evaluating the menus [35].

The snacks brought from home by the children were qualitatively assessed by the research team through observation. The evaluation of lunchboxes was carried out on a school day in the classroom, before the students were released for the break designated for school meals. The researchers worked in pairs and, with the support of the teachers responsible for the classes, asked the students to open their lunchboxes on their desks. Next, the researchers took photographic records of the contents of the lunchboxes, documenting all foods and beverages present.

A form was prepared for the tabulation of these data and later, based on the images obtained, the foods and beverages were identified and classified according to the degree of processing: unprocessed or minimally processed, processed, and ultra-processed, based on the classification of the Dietary Guidelines for the Brazilian Population [36]; namely, G1—“unprocessed or minimally processed foods”, G3—“processed foods”, and G4—“ultra-processed foods” [36].

The evaluation of foods available for sale in schools was based on interviews with the manager responsible for each school in order to understand how the cafeteria operated and what foods were available for sale, in addition to conducting sporadic visits to schools during recess to observe and record the foods sold.

### 2.4. Data Collection in the School Food Environment

A pilot study was conducted in a neighboring city to test the applicability, identify possible problems in the research methodology, and improve the training of the research team. The study allowed for necessary adjustments to be made to possible flaws and ambiguities in the questions, in addition to clarifying the instructions and the effectiveness of the collection instruments.

After the pilot study, data collection began in the municipality under study. A 400 m buffer was delineated from the main access gate of the schools to define the school surroundings, and all food retail establishments within this radius were audited.

During data collection in the school surroundings, it was found that the distance traveled by students to school was significantly longer than the initially defined 400 m area, which also had a reduced number of retail establishments. Thus, the community food environment (retail food establishments in the urban center of the municipality) was also surveyed, audited, and evaluated. The study area covered the urban center of the city, delimited according to the 2022 IBGE census map [37], including all food establishments in this area.

#### 2.4.1. Food Environment Audit

During visits to the establishments, the food environment assessment tools called ESAO-Store (ESAO-S) and ESAO-Restaurant (ESAO-R) were applied, as proposed by the São Paulo Obesogenic Environment Study (ESAO-SP) [38]. These tools consist of questions that aim to characterize the set of factors that can influence food choices. The tools were adapted to digital format using Google Forms, so that data tabulation occurred automatically in an Excel spreadsheet as soon as the data was entered and submitted online. The digital adaptation did not alter the content of the questions (only the format), and there was no loss of validity.

The ESAO-R tool was applied in establishments that sell food for immediate consumption and aims to evaluate items related to the availability and pricing of healthy and unhealthy foods, facilitators and barriers to healthy eating at restaurants, availability of nutrition information near the point of purchase or on the menu, and the presence of food marketing on the menu. The ESAO-S, on the other hand, is applied in establishments that sell food for consumption at the household level and assesses aspects related to food availability, variety, quality, pricing, signage, and promotional sales [38].

#### 2.4.2. Assessment of the Food Environment

Two methods were used to construct the food environment analysis indicators, which enabled the classification of food establishment types and the assessment of access to healthy foods.

The first method was based on the Locais-Nova classification system, which categorizes the types of food establishments according to the Dietary Guidelines for the Brazilian Population. Based on this approach, establishments were classified and counted according to the sixteen food purchase locations defined by the system. Subsequently, the businesses were grouped according to the three categories created by the Locais-Nova classification system in accordance with the recommendations of the Dietary Guidelines for the Brazilian Population; namely, sources of unprocessed or minimally processed foods and culinary ingredients, sources of processed foods, and sources of UPFs [39].

The second method was based on analyzing the quality of food offerings within establishments. Access to healthy foods was measured using assessment indices obtained from ESAO-S and ESAO-R data; namely, the Healthy Food Store Index (HFSI) for foods sold for consumption at the household level, and the Healthy Restaurant Index (HMRI) for foods sold for immediate consumption.

The HFSI, applied to ESAO-s, ranges from 1 to 16 points and assesses the availability, variety, and presence of advertising or promotional sales of healthy items (such as fruits and vegetables) and ultra-processed products (such as sugary drinks, corn snacks, and chocolate-filled cookies). In turn, the HMRI, used for ESAO-r, ranges from 0 to 8 points and considers the availability and promotional sales of vegetables, fruits, and fresh fruit juices, in addition to the presence of advertising for ultra-processed foods [40]. The interpretation of the index results is that the lower the score, the more limited the access to healthy foods in that establishment or in the area evaluated.

#### 2.4.3. Delimitation of the Study Area, Georeferencing, and Kernel Map

The study area was delimited using Google Maps 24.x, and the addresses of establishments selling food were georeferenced using the Global Positioning System (GPS) tool for the purpose of marking geographical points. The geographic coordinates, latitude and longitude, were obtained using the My GPS Coordinates application. These were exported to the Google Earth PRO tool for the purpose of marking geographic points and subsequently to the Qgis 3.40.9 software, which was used to create Kernel maps based on three variables: X (east coordinate), Y (south coordinate), and type of establishment, with a basic parameter of a 1000 m radius of influence for each point.

Kernel estimation is a nonparametric, exploratory statistical interpolation technique that reveals the spatial distribution pattern of points, generating a density surface that allows the visual identification of areas with higher intensity of event occurrence [41]. The Kernel Map was used to demonstrate, through a visual cluster detection test, the types of food establishments according to the Locais-Nova classification, as well as the location of schools. Food establishments were marked on the map according to the following legend: Type 1—sources for acquisition of unprocessed or minimally processed foods and culinary ingredients (green); Type 2—sources for acquisition of processed foods (yellow); Type 3—sources for acquisition of UPFs (red) [39].

### 2.5. Statistical Analyses

The data were organized using Microsoft Excel and then analyzed using descriptive statistics to characterize the sample, calculating the absolute and relative frequencies of the collected data using SPSS software, version 25.

The data were described so that the categorical variables are presented by absolute (n) and relative (%) frequency distribution. The normality of the data was confirmed by the Shapiro–Wilk (ESAO indices) and Kolmogorov–Smirnov (anthropometric data) tests. The comparison of anthropometric assessment data and food environment indices across schools was performed using the Mann–Whitney test for independent samples, as they presented a nonparametric distribution. The level of statistical significance adopted was 95% (*p* ≤ 0.05).

## 3. Results

### 3.1. Assessment in the Internal School Environment

#### 3.1.1. Anthropometric Assessment of Students

Table 2 presents the descriptive statistics of the variables analyzed in this study, including the minimum and maximum values observed, both for the total group of students and for each school evaluated.

The total sample consisted of 266 schoolchildren, 56% female and 44% male, aged between 5 and 11 years, enrolled in grades 1 to 4 of primary school.

The analysis revealed that there was no statistically significant difference between schools A and B in the variables of weight, height, body mass index, gender, and BMI/A categorization. The age variable showed a statistically significant difference (*p* = 0.029 *) between the schools, with a higher median in school B.

The median weight, height, and BMI of the students were 26.5 kg, 1.3 m, and 16.1 kg/m^2^, respectively, with the majority of children classified as normal weight (65.4%). The BMI-for-age categorization showed that 33.1% of schoolchildren in the municipality of JM had excess weight (overweight and obesity).

#### 3.1.2. Assessment of the Availability of Food and Beverages Offered to Schoolchildren

**Figure 1:**
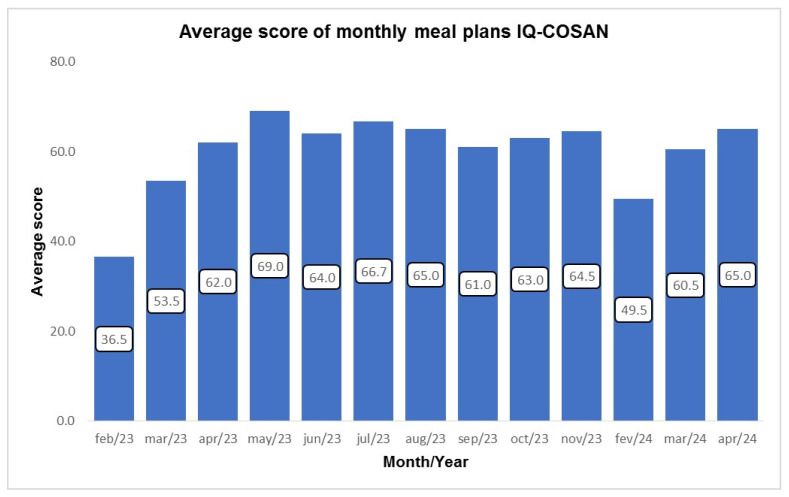
Mean score of monthly menus evaluated by IQ-COSAN.

The theoretical menu offered by the PNAE was evaluated based on the average scores assigned to each month analyzed, covering the period from February to November 2023 and the months of February, March, and April 2024. According to the tool’s classification scale, in February 2023, the menu was considered “Inadequate” (0 to 45.9 points), while in the remaining months, it scored in the “Needing improvement” category (46 to 75.9 points) (Figure 1).

Table 3 shows the total number of lunchboxes analyzed, by school, and the characteristics of the foods carried in the lunchboxes. Only the lunchboxes of students who voluntarily allowed access were analyzed, resulting in a sample of 243 schoolchildren.

The analysis revealed that 105 (43.2%) students brought snacks from home, with 54 (38.3%) students from school A and 51 (50%) from school B, with no significant difference between them (*p* = 0.069). Considering the group of students who brought lunchboxes to school, it was found that 5 (4.8%) brought U or MP foods, 85 (81%) brought P or UP foods, and 15 (14.3%) brought both categories.

With regard to food sales within schools, it was observed that there is no designated physical space characterized as a canteen for the display and sale of different types of food. It was observed that during the school break, a temporary food sales point is set up near the cafeteria, where an employee displays the food available for sale that day on a table. At the same time, an adapted room contains appliances such as a refrigerator and a microwave oven, which are used for the storage and reheating of savory snacks.

Furthermore, during the visits, it was found that School A sold ultra-processed yogurt ice cream, chicken and cheese pizza, and handmade popsicles, while School B sold the same ice cream, baked chicken snacks, baked ham and cheese snacks, and cakes prepared at the school itself.

### 3.2. Assessment of the School Environment

With regard to the categorization of food businesses, according to the Locais-Nova classification, six food retail establishments were found in the surroundings of both School A and School B (Table 4 and Table 5). However, in the surroundings of School A, there were four establishments in category 3 (sources for acquisition of ultra-processed foods) and in School B, there were three establishments in this category and one in category 2 (sources for acquisition of processed foods).

With regard to the HFSI, the median score among the schools was 6.0 points, with only one establishment in the surroundings of School A achieving this same score, while the median score for the two establishments in the surroundings of School B was 6.5 points (Table 6). Regarding the HMRI, the median SFE score among the schools was 1.0 point, and both schools had four establishments in their surroundings. The median score for establishments around School A was 1.5 points, and for School B, it was 1.0 point, with no statistically significant difference (*p* = 0.741) between schools (Table 7).

### 3.3. Assessment of the Community Food Environment

The study identified a total of 85 food establishments in the urban center of the municipality of JM, including 23 bars, 21 snack bars, 8 mini-markets and grocery stores, 12 restaurants, 11 fruit and vegetable stores, 6 bakeries and pastry shops, 5 supermarkets, one convenience store, one candy store, and one butcher shop, described according to the Local-Nova establishment group (Table 8).

Regarding the classification of food establishments according to the recommendations of the Dietary Guidelines for the Brazilian Population based on the Locais-Nova study, it was found that 60 (70.6%) of the establishments in the municipality fall into category 3 of the Locais-Nova classification. This category corresponds to establishments that predominantly sell ultra-processed foods. In addition, 6 (7%) fall into category 2 (Sources for acquisition of processed foods) and 19 establishments (22.4%) are part of category 1 (Sources for acquisition of unprocessed or minimally processed foods and culinary ingredients) (Table 9). Supermarkets, bars, and convenience stores that have more than one classification according to the Locais-Nova study were classified in this study as sources for acquisition of ultra-processed foods due to the predominance of these types of foods in these establishments in the city.

The medians of the assessment indices of access to food in food retail establishments for immediate consumption (HMRI) and at the household level (HFSI) were 2.0 and 8.0 points, respectively. These results presented low indices of access to food for immediate consumption and at the household level.

**Figure 2:**
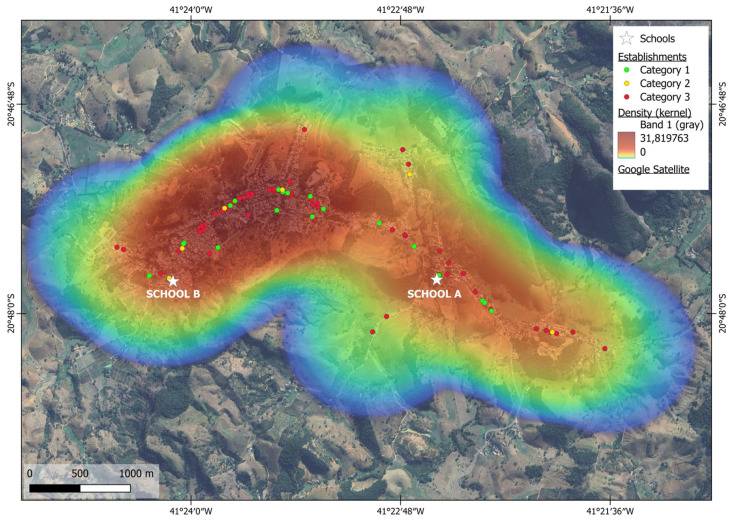
Kernel map of food establishments in the urban center of JM/ES according to the Locais-Nova classification.

The Kernel map, generated from this research, shows the distribution of commercial establishments around school areas and in the urban center of the municipality, with the heat map varying from shades of blue (low density) to red (high density). The areas shown in red on the map represent a higher concentration of visited commercial establishments, whereas the areas in blue indicate low or no concentration of commercial establishments (Figure 2). Visual analysis reveals that the central regions of the city have a higher density of commercial establishments, as evidenced by the red/orange colors. In contrast, the other areas are predominantly residential and vegetated, presenting a lower commercial density, which is reflected by the greener/bluish coloration on the map.

## 4. Discussion

This study evaluated the anthropometric indicators of schoolchildren and the school and community food environment in Jerônimo Monteiro, a small-sized rural Brazilian municipality. This study identified considerable results of schoolchildren with excess weight, inadequacies in the supply of food and beverages for schoolchildren, and a predominance of establishments selling ultra-processed foods compared to those offering healthy foods outside schools.

There was no significant difference in the variables of weight, height, body mass index, sex, and BMI/A categorization between schools A and B, revealing the homogeneity and representativeness of the sample in relation to the population of students in the municipal school system from years 1 to 4.

This result can be explained by the fact that the municipality is considered small-sized and has a relatively small land area (177.342 km^2^), according to the IBGE classification [32]. The similarity of the sample was also evidenced by the results of the 2021 *Índice de Desenvolvimento da Educação Básica* (IDEB–Basic Education Development Index), which presented similar values between schools, corresponding to 6.2 in School A and 5.9 in School B [42]. Given this, considering the homogeneity of the sample, the results are presented for the total group of schoolchildren, without segmentation by school.

The assessment of children’s nutritional status using anthropometric indicators is a relevant tool recommended by the Brazilian Ministry of Health to outline nutritional profiles and identify situations of nutritional risk. In this study, the results of this assessment, based on the interpretation of BMI/A, revealed a prevalence of 33.1% of excess weight (overweight and obesity) among schoolchildren in JM. This percentage reveals a worrying reality of nutritional risk for a small municipality, which is close to the statistics on childhood overweight and obesity revealed in Brazil in recent years [43].

According to the consolidated nutritional status report for 2024 by the Food and Nutrition Surveillance System (SISVAN), 29.8% of Brazilian children between the ages of 5 and 10 presented excess weight, and for the state of Espírito Santo, this percentage is 29.2% [44]. A study conducted with schoolchildren aged 7 to 10 years in a public school in the municipality of Lagoinha (SP) found that 20.2% of students had excess weight [45]. These results show lower percentages than those found for excess weight in the present study. Additionally, like JM, this city is also considered small-sized, with a land area of 255.472 km^2^ and an estimated population of 5083 inhabitants according to the IBGE [46].

Regarding the assessment of the availability of different food items offered to schoolchildren, it was found that the school meal menu needs improvement and that most students who bring lunchboxes are exposed to ultra-processed foods.

The data identifying 81% of processed foods and UPFs present in lunchboxes is consistent with the reports obtained in interviews with school principals. The managers reported that, based on daily observation of recesses, they always noticed the predominance of UPFs among the items brought by students. A study conducted in the municipal school system of the city of Caxias do Sul-RS found a prevalence of 69.7% daily consumption of UPFs among schoolchildren. The results also showed that consumption of these products was higher among students who brought snacks from home or purchased food at school [4].

Furthermore, although we did not find physical canteen structures in the municipality’s schools, we did find the sale of food preparations containing ultra-processed ingredients as well as the direct sale of UPFs. The *Fundo Nacional de Desenvolvimento da Educação* (FNDE—National Fund for Education Development), based on Technical Note No. 2974175/2022 [47], establishes general guidelines regarding the sale of food in public elementary schools covered by the PNAE, emphasizing that these locations should encourage the consumption of unprocessed and minimally processed foods and restrict the sale of ultra-processed foods. At the municipal level, Ordinance No. 052/2011 establishes rules for the operation of school canteens in the municipal education network in line with the principles defined by the FNDE. In addition, the country has Decree No. 11,821, of December 2023, which provides guidelines that guide the promotion of adequate and healthy eating in the school environment. However, school administrators still lack more specific guidelines regarding the types of food that can be sold.

The sale of UPFs within schools is a reality found in different regions of Brazil, and this supply is associated with higher consumption of these products [4,48,49]. UPFs contain ingredients such as additives and preservatives, which give them intense flavor and long shelf life, in addition to being rich in sugars, fats, and sodium. These constituents make these products highly palatable, high in energy, and low in nutritional value, exposing consumers to adverse health outcomes and increasing the risk of developing chronic diseases, including obesity [50].

Evidence confirms the risks associated with early and frequent consumption of UPFs, and this intake contradicts the recommendations of competent bodies for healthy eating [50,51,52]. The Brazilian Ministry of Health reiterates that children’s diets should be based on the consumption of unprocessed and minimally processed foods and follow qualitative and quantitative recommendations that ensure the availability of the nutrients necessary for good health, proper functioning of the body, and full development [53].

Schools, as fundamental spaces for education, knowledge building, and habit development, should play a central role in promoting adequate eating habits among children. In Brazil, the PNAE is one of the government initiatives that aims to provide meals universally to public schools, covering the nutritional needs of students during the school year, contributing to their biopsychosocial development, academic performance, and the formation of healthy eating habits. The PNAE, recognized as the largest school feeding program in the world, is a major ally in protecting and promoting school health [54,55].

In this context, IQCOSAN stands out as an important strategic tool integrated into the PNAE, which allows for monitoring and evaluating the program’s implementation by assessing school menus prepared by states and municipalities. This tool allows for identifying critical issues and proposing improvements, ensuring that schools offer meals that are in line with public food and nutrition policy recommendations.

In a study conducted by Mendes [56], three months of menus from daycare centers and elementary schools in the public school system of the state of São Paulo were evaluated using the IQ COSAN tool. Similarly to the results of this study, most of the menus analyzed in the study were classified in the “Needing improvement” category, with a mean score of 72.05. These findings show that, although the menus partially comply with the PNAE guidelines, they still have significant limitations that need to be overcome.

It is also worth noting that most of the studies found in the literature are based on scores referring to the analysis of only one month of menus, unlike the present study, which chose to evaluate 13 months in order to obtain a more realistic picture of the food that schoolchildren in the municipality of JM are exposed to [57,58,59].

Regarding the results of the food environment in the school surroundings, it was found that, in this municipality, the school factor is not considered a predictor of commercial concentration, since only six food establishments were identified within the 400 m buffer of both schools. These results do not reflect the reality of the distribution of establishments around schools as found in studies conducted in large cities [60,61,62,63].

It was observed that the city’s commercial establishments, as well as banks, hospitals, health centers, courthouses, and pharmacies, are located in the area referred to as the “city center” by local residents and belonging to the area defined as the urban center by the IBGE. Thus, commercial activity in the municipality is concentrated in this region, where residents must travel daily to meet their essential needs, carry out their daily activities, and satisfy their consumption needs. Another important point observed during the data collection was that the distance traveled by students to school was actually longer than the 400 m area initially defined.

Therefore, according to this reality found in the municipality, it is plausible that the community food environment (central region of the municipality) be considered the food environment to which schoolchildren are frequently exposed. Therefore, the data found in this study allow us to infer that schoolchildren in JM are inserted in a food environment with obesogenic characteristics that favors the consumption of UPFs, since 70.5% of food retail establishments in the municipality are sources of ultra-processed foods. In addition, the values found for the indices assessing access to food in establishments selling food for immediate consumption (HMRI) and at the household level (HFSI) are considered low, which points to compromised access to the marketing of healthy foods.

The mapping of the types of food establishments, according to the Locais-Nova classification, based on Kernel’s estimate, allows a visualization of the distribution of these establishments around schools and in the urban center of the municipality (Figure 2). The analysis shows a greater predominance of type 3 establishments (sources for acquisition of ultra-processed foods), represented by red dots, compared to type 1 establishments (sources for acquisition of unprocessed or minimally processed foods and culinary ingredients) and type 2 establishments (sources for acquisition of processed foods), represented by green and yellow dots, respectively.

The analysis also shows that the central areas of the municipality have a higher density of commercial establishments, highlighted by the red/orange shades on the map. In contrast, the areas surrounding schools have a reduced number of these points of sale. In line with the findings of this study, Novaes 2018 [64] revealed in a study conducted throughout the school district of Viçosa-MG that establishments were concentrated in more central regions, mainly due to economic development.

Food establishments around schools, including public spaces that may or may not promote healthy eating, are part of the school food environment and have a significant impact on food choices, directly influencing the health and nutritional status of individuals [65]. According to De Castro and Canella [66], these environments are strategic locations for intervention because they bring together groups of people and offer opportunities for regulating and promoting healthy food choices. Thus, it is plausible that schools take an active role in nutritional governance, whether through the regulation of cafeterias, the definition of rules for lunchboxes, or the promotion of food and nutrition education.

However, the findings of this study highlight the need for urgent regulatory actions to reverse this environmental scenario, which require broad and intersectoral strategies involving the joint work of federal, state, and municipal agencies, as well as the participation of civil society.

Among the strengths of this study is the fact that it was conducted in a small-sized municipality, a context that has been little evaluated in the scientific community. Another highlight was the joint assessment of the food environment inside and outside schools, together with the anthropometric assessment of schoolchildren based on primary data for both assessments.

Despite these points, the study has some limitations. As it is a cross-sectional design, it is not possible to establish causal relationships between the variables analyzed. In addition, socioeconomic and individual data on food consumption, behaviors, and food choices in the home environment were not evaluated. These points restrict a broader understanding of factors that also influence the development of overweight and obesity.

## 5. Conclusions

This study showed that the municipality of JM has a predominance of establishments that mainly sell ultra-processed foods, which exposes local schoolchildren to an obesogenic food environment. Moreover, the results showed high rates of overweight and obesity among schoolchildren, high consumption of ultra-processed foods, and the need for improvements in the quality of the menus offered by the municipality.

The predominant presence of ultra-processed foods in the school food environment is one of the factors that influence the development of diseases such as obesity. Therefore, it is necessary to strengthen public policies that encourage the supply of healthy foods in the surroundings of schools, aiming to promote a more balanced diet and improve the health conditions of the student population of Jerônimo Monteiro.

## Figures and Tables

**Table 1:** Data collection schedule.

Month/Year	Activity Conducted
2023	Presentation of the project to the Municipal Department of Education of Jerônimo Monteiro and completion of the legal procedures.
February to March/2024	Presentation of the project to the school administration and visits to the schools in order to become familiar with and observe the school routine.
April/2024	Application of the Free and Informed Consent Form (TCLE) to the children’s legal guardians. Application of the Free and Informed Assent Form (TALE) to the children.
May to June/2024	Anthropometric assessment of the students. Assessment of the availability of foods and beverages offered to schoolchildren through the evaluation of lunchboxes and foods sold in schools.
June to July/2024	Data collection in the surroundings of the school food environment.
July to November/2024	Data collection in the community food environment.

**Table 2:** Characterization of the sample and classification of the nutritional status of schoolchildren by school.

Characteristic	Total	School A	School B	*p*-Value
Total	266			
Age (years)	7 (5–10)	7 (5–9)	8 (6–10)	*p* = 0.029 *
Gender				*p* = 0.64
Female	149 (56%)	90 (60.4%)	59 (39.6%)	
Male	117 (44%)	74 (63.2%)	43 (36.7%)	
Weight (kg)	26.5 (15.6–67.4)	25.7 (15.6–64.7)	27.2 (17.4–67.3)	*p* = 0.17
Height (m)	1.3 (1.1–1.5)	1.3 (1.1–1.5)	1.3 (1.1–1.5)	*p* = 0.96
BMI (kg/m^2^)	16.1 (12.5–32.5)	16.0 (12.8–32.5)	16.4 (12.5–32.0)	*p* = 0.22
BMI/A Z-score	0.27 (−2.92–5.73)	0.21 (−2.4–4.5)	0.4 (−2.9–5.7)	*p* = 0.39
BMI/A categorization				*p* = 0.35
Thinness	4 (1.5%)	3 (1.8%)	1 (1.0%)	
Normal weight	174 (65.4%)	109 (66.5%)	65 (63.7%)	
Overweight	41 (15.4%)	26 (15.9%)	15 (14.7%)	
Obesity	47 (17.7%)	26 (15.8%)	21 (20.6%)	
Excess weight	88 (33.1%)	52 (31.7%)	36 (35.3%)	

Variables expressed as frequency and percentage or median, minimum, and maximum. Mann–Whitney and Chi-square tests. * Statistical significance, *p* < 0.05. Source: Authors’ preparation.

**Table 3:** Evaluation of lunchboxes according to schools.

Characteristics	Total % (n = 243)	School A % (n = 141)	School B % (n = 102)	*p* Value
Presence of Lunchbox				*p* = 0.069
Yes	43.2 (105)	38.3% (54)	50 (51)	
No	56.8 (138)	61.7 (87)	50 (51)	
Food classification	Total % (n = 105)	School A % (n = 54)	School B % (n = 51)	*p* = 0.866
U/MP foods	4.8 (5)	5.6 (3)	3.9 (2)	
P or UP foods	81 (85)	81.5 (44)	80.4 (41)	
U/MP and P/UP foods	14.3 (15)	13	15.7 (8)	

Statistical significance, *p* < 0.05. U: Unprocessed; MP: Minimally Processed; P: Processed; UP: Ultra-processed. Source: Author.

**Table 4:** Places for acquisition of food according to the Locais-Nova classification, within a 400 m buffer zone of School A (n = 6).

Classification	Description	Type of Establishment	Total
1	Sources for acquisition of unprocessed or minimally processed foods and culinary ingredients	Restaurant Fruit and vegetable market	2
2	Sources for acquisition of processed foods	-	0
3	Sources for acquisition of ultra-processed foods	Bar Supermarkets	4

Source: Authors’ preparation.

**Table 5:** Places for acquisition of food according to Locais-Nova classification, within a 400 m buffer zone of School B (n = 6).

Classification	Description	Type of Establishment	Total
1	Sources for acquisition of unprocessed or minimally processed food and culinary ingredients	Restaurant Fruit and vegetable market	2
2	Sources for acquisition of processed foods	Bakery	1
3	Sources for acquisition of ultra-processed foods	Bar Grocery stores	3

**Table 6:** Scores for the Healthy Food Store Index (HFSI), which assesses access to food in stores selling food for home consumption, by school.

SFE *	School A (n = 1)	School B (n = 2)
Median index (min; max)	Index **	Mean index
6.0 (4;9)	6.0	6.5

* SFE: School Food Environment. ** Only one establishment. Source: Authors’ preparation.

**Table 7:** Scores for the Healthy Restaurant Index (HMRI), which assesses access to food in establishments selling food for immediate consumption, by school unit.

SFE *	School A (n = 4)	School B (n = 4)	*p* Value
Median index	Median index	Median index	*p* = 0.741
1.0 (1;4)	1.5 (1;3)	1.0 (1;4)	

* SFE: School Food Environment. Source: own work.

**Table 8:** Description of food purchase establishments in JM/ES, 2025.

Grocery Store Groups	Total Per Group
Bars	23
Snack bars	21
Mini-markets and grocery stores	8
Restaurants	8
Fruit and vegetable stores	6
Bakeries and pastry shops	6
Supermarkets	5
Orange stand	4
Convenience stores	2
Candy stores	1
Butcher shops	1
Total number of establishments	85

Note: The other groups were not included in the table because they were not present in the municipality.

**Table 9:** Description of the food environment. Places for acquisition of food according to Locais-Nova based on the recommendations of the Dietary Guidelines for the Brazilian Population of JM/ES, 2025 (n = 85).

Classification	Description	Type of Establishment	Total
1	Sources for acquisition of unprocessed or minimally processed foods and culinary ingredients	Restaurants (8) Fruit and vegetable markets (6) Orange stands (4) Butcher shop (1)	19
2	Sources for acquisition of processed foods	Bakeries	6
3	Sources for acquisition of ultra-processed foods	Bar (23) Snack bars (21) Grocery stores (8) Supermarkets (5) Convenience stores (2) Candy stores (1)	60

Source: Authors’ preparation.

## Data Availability

The datasets presented in this article are not readily available because the data in this study constitute preliminary results from an ongoing study in the state of Espírito Santo. Requests for access to the datasets should be directed to raisapessini@gmail.com and miriam.rodrigues@ufes.br.

## Abbreviations

The following abbreviations are used in this manuscript:

UPFs	Ultra-processed foods
FAO	Food and Agriculture Organization of the United Nations
JM	Jerônimo Monteiro
ES	Espírito Santo
UFES	Universidade Federal do Espírito Santo
IBGE	Brazilian Institute of Geography and Statistics
BMI	Body Mass Index
WHO	World Health Organization
PNAE	National School Feeding Program
IQ COSAN	Food and Nutrition Security Coordination Quality Index
ESAO-SP	Study of the Obesogenic Environment in São Paulo
ESAO-S	ESAO-Store
ESAO-R	ESAO-Restaurant
HFSI	Healthy Food Store Index
HMRI	Healthy Market Retail Index
GPS	Global Positioning System
BMI/A	Body Mass Index for age
IDEB	Basic Education Development Index
SISVAN	Food and Nutrition Surveillance System
